# Mortality and years of life lost due to breast cancer attributable to physical inactivity in the Brazilian female population (1990–2015)

**DOI:** 10.1038/s41598-018-29467-7

**Published:** 2018-07-24

**Authors:** Diego Augusto Santos Silva, Mark Stephen Tremblay, Maria de Fatima Marinho de Souza, Maximiliano Ribeiro Guerra, Meghan Mooney, Mohsen Naghavi, Deborah Carvalho Malta

**Affiliations:** 1 Federal University of Santa Catarina, Research Center in Kinanthropometry and Human Performance, Florianopolis, SC 88040-900 Brazil; 2 Children’s Hospital of Eastern Ontario Research Institute, Ottawa, ON ONK1H5B2 Canada; 3 Ministry of Health, Department of Surveillance of Noncommunicable Diseases, and Injuries, and Health Promotion, Brasília, DF 70058-900 Brazil; 4 Federal University of Juiz de Fora, Post-graduate Program in Public Health, Juiz de Fora, MG 36036-330 Brazil; 5 Institute for Health Metrics and Evaluation, Seattle, WA 98121 USA; 6 Federal University of Minas Gerais, Department of Maternal and Child Nursing and Public Health, School of Nursing, Belo Horizonte, MG 31270-901 Brazil

## Abstract

The aims of this study were as follows: to estimate the mortality and years of life lost, assessed by disability-adjusted life years (DALYs), due to breast cancer attributable to physical inactivity in Brazilian women; to compare the estimates attributable to physical inactivity and to other modifiable risk factors; and to analyse the temporal evolution of these estimates within Brazilian states over 25 years (1990–2015), compared with global estimates. Databases from the Global Burden of Disease Study for Brazil, Brazilian states, and other parts of the world were used. Physical inactivity has contributed to a substantial number of deaths (1990: 875; 2015: 2,075) and DALYs (1990: 28,089; 2015: 60,585) due to breast cancer in Brazil. Physical inactivity was responsible for more deaths and DALYs (~12.0%) due to breast cancer than other modifiable risk factors (~5.0%). The Brazilian states with better socioeconomic indicators had higher age-standardized rates of mortality and morbidity due to breast cancer attributable to physical inactivity. From 1990 to 2015, mortality due to breast cancer attributable to physical inactivity increased in Brazil (0.77%; 95%U.I.: 0.27–1.47) and decreased (−2.84%; 95%U.I.: −4.35 – −0.10) around the world. These findings support the promotion of physical activity in the Brazilian female population to prevent and manage breast cancer.

## Introduction

Breast cancer is a disease more common in the female population, and in 2017, it was the leading cause of death among females around the world^1–3^. Breast cancer was also responsible for substantial years of life lost around the world, becoming a type of cancer that, in addition to high mortality, is also responsible for early morbidity^1–3^. This neoplasm has multifactorial aetiology that includes both genetic and modifiable lifestyle factors^1,2^. Among the lifestyle factors, physical inactivity, obesity, inadequate diet and excessive alcohol use stand out as modifiable risk factors, which, if avoided, could help in the prevention and management of breast cancer^1,2^. It is not known whether studies have investigated the burden of breast cancer mortality due to all of these modifiable risk factors in the same population.

Physical inactivity appears to reduce the risk of breast cancer through a number of mechanisms, including body fat reduction, which in turn reduces oestrogen and insulin concentrations. Indeed, these hormones have mitogenic effects on mammary cells^3,4^. Likewise, leptin released from adipose tissue, which has been associated with post-menopausal breast cancer, significantly decreases following aerobic activity^2–4^. Therefore, physical activity may have positive effects on reducing the incidence of breast cancer by modifying endocrine function and improving the immune system^5^. A systematic review and meta-analysis on the association between physical inactivity and chronic disease outcomes found a strong correlation between physical inactivity and the risk of five chronic diseases, including breast cancer^6^. In addition, an aggregation of 35 prospective cohort studies reported a 14% reduction in the risk of breast cancer for physically active individuals^6^.

There are short and persistent adverse, even toxic, effects of breast cancer treatment that might be prevented, attenuated, treated, or rehabilitated through regular physical activity^7,8^. Breast cancer patients, who exercised while undergoing cancer treatments, were not only able to tolerate the exercise prescriptions but also presented a more favourable trend towards the alleviation of decrements in functional capacity, fatigue levels, and depression when compared to patients who did not exercise. Additionally, and very importantly, no adverse events were reported, and patients seemed to have no problems engaging in regular physical activity^7,8^. In this sense, physical activity is also important in the tertiary prevention of breast cancer.

Studies analysing data on the global burden of breast cancer mortality and morbidity have found no association with a single specific cause but with several causal factors of the disease^1,9–11^. The information on the burden of mortality or morbidity of a disease due to several causes is important because it provides essential data for the epidemiological monitoring of the disease. However, it does not allow for the identification and quantification of causal chain risk factors that impact the burden of disease^12^. The study of physical inactivity as a specific risk factor compared with other modifiable risk factors in the global burden of mortality and morbidity of breast cancer may be useful for public policies to intervene not only related to the disease but also related to associated risk factors, which may result, in the medium and long term, in lower incidence of the disease and better quality of life of the affected population^12^.

Brazil is a middle-income country that has recently improved its national health databases and implemented a series of surveys to better assess noncommunicable diseases, risk factors, prevalence and disease burden^13,14^. The results of these national health databases showed that there is an inequality in the burden of diseases and risk factors among the states of Brazil^13,14^. This inequality can be reflected by socioeconomic differences and the level of development of each of the Brazilian regions. In this sense, the investigation of the evolution of breast cancer mortality and morbidity due to physical inactivity in the Brazilian states over 25 years may be useful for health policies.

The aim of this study was to estimate the mortality and years of life lost, assessed by disability-adjusted life years (DALYs), due to breast cancer attributable to physical inactivity in the female population from Brazil and Brazilian states. We also aimed to compare the estimates of mortality and DALYs due to breast cancer attributable to physical inactivity with estimates attributable to other modifiable risk factors. Finally, we aimed to analyse the temporal trends of the estimates of mortality and DALYs due to breast cancer attributable to physical inactivity over 25 years (1990–2015) in comparison with global estimates and according to the socioeconomic status of Brazilian states.

## Results

### Incident breast cancer cases

In 1990 and in 2015, there were 983,231 (95%U.I.: 925,585–1,063,992) and 2,377,627 (95%U.I.: 2,236,222–2,496,759) incident breast cancer cases worldwide, respectively. In Brazil, 26,558 (95%U.I.: 22,328–29,349) incident breast cancer cases were estimated in 1990 and 79,015 (95%U.I.: 66,251–91,561) in 2015. These values represented, in 1990, an age-standardized rate of incident breast cancer cases (per 100,000 inhabitants) of 48.88 (95%U.I.: 46.10–52.72) around the world and of 54.73 (95%U.I.: 45.52–60.30) in Brazil. In 2015, the age-standardized rate of incident breast cancer cases was 65.49 (95%U.I.: 61.67–68.79) around the world and 74.02 (95%U.I.: 61.90–85.86) in Brazil. From 1990 to 2015, there was a large increase in the age-standardized rate of incident breast cancer cases around the world (39.72%; 95%U.I.: 29.39–49.95) and in Brazil (41.45%; 95%U.I.: 14.56–69.93). Table 1 provides detailed information on incident breast cancer cases (absolute number and age-standardized rate) for each Brazilian state.

**Table 1:** Number and age-standardized rate (per 100,000 inhabitants) of incident cases from breast cancer in women (≥15 years old) around the world, Brazil, and Brazilian states in 1990 and 2015.

Incident breast cancer cases															
	1990			2015			1990			2015			Change (1990–2015)		
	Cases	95% U.I.		Cases	95% U.I.		Rate*	95% U.I.		Rate*	95% U.I.		%*	95% U.I.	
Global	983,231	925,585	1,063,992	2,377,627	2,236,222	2,496,759	48.88	46.10	52.72	65.49	61.67	68.79	39.72	29.39	49.95
Brazil	26,558	22,328	29,349	79,015	66,251	91,561	54.73	45.52	60.30	74.02	61.90	85.86	41.45	14.56	69.93
Acre	29	24	43	137	105	183	30.16	24.62	43.88	52.43	40.30	69.65	85.20	35.91	147.49
Alagoas	287	243	344	890	688	1,137	39.70	33.58	47.66	60.16	46.58	76.70	61.46	20.81	118.25
Amapá	15	12	24	96	65	156	23.63	18.38	38.77	42.28	28.57	68.63	89.12	27.58	176.91
Amazonas	178	147	214	827	633	1,073	39.57	32.94	47.03	64.18	49.95	82.06	72.17	25.33	138.62
Bahia	1,525	1,276	1,790	5,064	3,915	6,426	43.88	36.76	51.13	69.20	53.47	87.14	64.22	23.25	117.98
Ceará	947	735	1,122	3,302	2,406	4,146	45.86	35.90	53.97	77.22	56.48	96.99	77.31	33.35	133.05
Distrito Federal	253	217	289	1,023	775	1,306	55.65	47.66	63.34	69.54	52.59	88.05	31.66	−3.41	72.87
Espírito Santo	379	329	445	1,363	1,077	1,701	46.30	40.29	54.03	66.09	52.38	82.21	48.65	11.76	94.29
Goiás	520	445	633	2,002	1,614	2,467	45.01	38.73	54.05	62.73	50.28	77.11	43.51	9.67	85.19
Maranhão	442	330	570	1,551	1,106	2,128	33.50	24.96	42.98	57.77	41.49	79.50	77.13	19.04	165.29
Mato Grosso	182	148	223	877	667	1,108	39.78	32.83	48.58	63.00	48.38	79.04	64.40	16.98	124.98
Mato Grosso do Sul	240	203	283	866	664	1,095	47.24	40.64	55.28	67.51	51.91	85.06	49.74	12.20	95.13
Minas Gerais	2,663	2,305	3,075	7,991	6,382	9,863	50.68	43.62	58.28	68.78	55.09	84.86	42.82	8.67	85.85
Paraná	1,417	1,227	1,639	4,551	3,610	5,678	52.17	45.32	60.28	73.76	58.59	91.72	47.15	10.31	90.04
Paraíba	446	379	522	1,369	1,025	1,769	41.02	34.88	47.94	68.07	51.33	87.86	72.21	25.32	134.66
Pará	440	358	535	1,674	1,229	2,224	36.40	29.93	44.10	56.62	41.83	74.62	60.47	16.98	124.39
Pernambuco	1,247	1,044	1,478	3,583	2,685	4,560	51.17	42.86	60.54	76.49	57.45	97.16	58.89	17.00	111.64
Piaui	258	207	318	934	707	1,197	35.16	28.34	43.69	61.46	46.67	78.82	78.59	30.95	139.93
Rio de Janeiro	3,988	2,722	4,559	9,820	7,087	11,971	73.70	49.76	84.02	93.43	67.18	113.94	32.88	3.47	66.66
Rio Grande do Norte	327	269	379	1,087	842	1,340	42.18	35.17	48.82	64.05	49.84	78.70	58.04	19.07	104.16
Rio Grande do Sul	2,477	1,849	2,856	6,097	4,545	7,876	68.15	50.41	78.57	84.07	62.41	108.35	28.48	−5.06	74.57
Rondônia	82	66	109	349	258	497	36.06	29.63	48.86	50.07	37.58	71.66	44.65	7.85	93.44
Roraima	15	12	18	95	75	117	45.57	36.95	52.60	63.42	49.90	78.30	47.66	15.14	92.92
Santa Catarina	749	634	868	2,671	2,053	3,415	52.92	44.52	60.68	72.82	55.90	92.27	43.74	5.95	93.62
Sergipe	192	162	225	710	551	909	42.95	36.20	49.98	70.64	55.00	89.17	76.61	28.62	137.40
São Paulo	7,192	5,703	8,078	19,767	15,714	23,834	63.05	49.17	71.15	77.42	61.38	93.32	28.26	−0.59	62.45
Tocantins	70	52	96	320	240	430	31.79	23.90	42.94	54.72	40.90	72.79	79.00	22.69	164.69

^*^Age-standardized rate; U.I.: uncertainty interval.

### Deaths and DALYs due to breast cancer

Around the world, 324,867 (95%U.I.: 312,956–347,331) deaths due to breast cancer due to all causes were estimated in 1990 and 523,487 (95%U.I.: 492,250–543,275) in 2015. In Brazil, 7,264 (95%U.I.: 6,185–7,694) deaths from breast cancer were estimated in 1990 and 16,964 (95%U.I.: 14,880–18,402) in 2015. Supplementary Table S1 provides the details on breast cancer mortality due to all causes (absolute number and age-standardized rate) for each Brazilian state.

Around the world, 9,578,973 (95%U.I.: 9,067,559–10,395,449) DALYs due to breast cancer attributable to all causes were estimated in 1990 and 15,137,828 (95%U.I.: 14,156,028–15,936,286) in 2015. In Brazil, 236,482 (95%U.I.: 205,941–251,674) DALYs were estimated in 1990 and 503,463 (95%U.I.: 443,581–548,345) in 2015. Supplementary Table S2 shows the information on breast cancer DALYs due to all causes (absolute number and age-standardized rate) for each Brazilian state.

Around the world, 63.265 (95%U.I.: 52.177–73.225) deaths due to breast cancer attributable to all risk factors (physical inactivity, alcohol use, high body mass index, diet high in sugar-sweetened beverages) were estimated in 1990 and 103,829 (95%U.I.: 84,505–123,222) in 2015. In Brazil, 1,212 (95%U.I.: 922–1,527) deaths from breast cancer were estimated in 1990 and 3,166 (95%U.I.: 2,359–4,149) in 2015. Supplementary Table S3 shows the information on breast cancer mortality due to all risk factors (absolute number and age-standardized rate) for each Brazilian state.

Around the world, 1,709,585 (95%U.I.: 1,427,601–1,987,977) DALYs due to breast cancer attributable to all risk factors (physical inactivity, alcohol use, high body mass index, diet high in sugar-sweetened beverages) were estimated in 1990 and 2,743,041 (95%U.I.: 2,257,795–3,240,611) in 2015. In Brazil, 35,113 (95%U.I.: 26,347–44,419) DALYs were estimated in 1990 and 82,164 (95%U.I.: 60,819–105,915) in 2015. Supplementary Table S4 shows the information on breast cancer DALYs due to all risk factors for each Brazilian state.

### Mortality and morbidity due to physical inactivity

Regarding all-cause mortality and DALYs due to physical inactivity, Supplementary Table S5 and Supplementary Table S6 shows the information on mortality and DALYs by all causes due to physical inactivity for each Brazilian state, respectively.

In relation to breast cancer mortality due to physical inactivity, 29,605 (95%U.I.: 21,397–37,982) deaths were estimated in 1990 and 46,720 (95%U.I.: 34,033–59,421) in 2015 around the world. These values represented an age-standardized rate of deaths per 100,000 inhabitants of 1.54 (95%U.I.: 1.11–1.97) in 1990 and 1.31 (95%U.I.: 0.95–1.66) in 2015. In Brazil, 875 (95%U.I.: 646–1,110) deaths were estimated in 1990 and 2,075 (95%U.I.: 1,528–2,646) in 2015, which represented an age-standardized mortality rate of 1.99 (95%U.I.: 1.46–2.52) in 1990 and 2.00 (95%U.I.: 1.47–2.54) in 2015. From 1990 to 2015, there was a decline in the age-standardized rate of deaths due to breast cancer attributable to physical inactivity (−2.84%; 95%U.I.: −4.35 – −0.10) around the world, while Brazil had an increase (0.77%; 95%U.I.: 0.27–1.47). Table 2 shows the information on mortality due to breast cancer attributable to physical inactivity for each Brazilian state.

**Table 2:** Number and age-standardized rate (per 100,000 inhabitants) of deaths from breast cancer due to physical inactivity in women (≥25 years old) around the world, Brazil, and Brazilian states in 1990 and 2015.

	Breast cancer mortality due to physical inactivity														
	1990			2015			1990			2015			Change (1990–2015)		
	Deaths	95% U.I.		Deaths	95% U.I.		Rate*	95% U.I.		Rate*	95% U.I.		%*	95% U.I.	
Global	29,605	21,397	37,982	46,720	34,033	59,421	1.54	1.11	1.97	1.31	0.95	1.66	−2.84	−4.35	−0.10
Brazil	875	646	1,110	2,075	1,528	2,646	1.99	1.46	2.52	2.00	1.47	2.54	0.77	0.27	1.47
Acre	01	01	01	04	02	05	1.14	0.80	1.66	1.46	0.96	2.07	0.38	−0.24	1.18
Alagoas	10	07	13	23	16	32	1.45	1.02	1.93	1.63	1.14	2.23	0.39	−0.30	1.23
Amapá	00	00	01	02	01	04	0.89	0.58	1.56	1.17	0.70	1.96	0.66	0.00	1.64
Amazonas	06	04	08	21	14	30	1.51	1.05	2.03	1.78	1.19	2.53	0.29	−0.38	1.12
Bahia	53	37	69	135	91	184	1.65	1.18	2.17	1.89	1.28	2.58	0.33	−0.35	1.11
Ceará	32	22	43	87	56	120	1.66	1.16	2.23	2.08	1.35	2.87	0.58	−0.14	1.53
Distrito Federal	08	05	10	27	18	37	2.03	1.42	2.64	1.86	1.27	2.57	0.64	−0.13	1.74
Espírito Santo	12	09	16	35	24	48	1.62	1.17	2.11	1.73	1.19	2.38	0.80	0.01	1.90
Goiás	17	12	22	52	35	71	1.65	1.18	2.19	1.72	1.18	2.34	0.46	−0.27	1.41
Maranhão	15	10	21	40	25	58	1.23	0.82	1.73	1.55	0.99	2.24	0.43	−0.27	1.38
Mato Grosso	06	04	08	23	15	31	1.51	1.06	2.03	1.76	1.21	2.40	0.42	−0.34	1.23
Mato Grosso do Sul	07	05	10	22	15	30	1.69	1.24	2.20	1.77	1.23	2.42	0.30	−0.52	1.37
Minas Gerais	87	63	112	207	145	278	1.81	1.31	2.34	1.81	1.27	2.43	0.40	−0.34	1.42
Paraná	45	32	57	117	80	162	1.87	1.35	2.42	1.97	1.34	2.70	0.83	0.04	1.88
Paraíba	16	11	21	37	25	52	1.52	1.08	1.99	1.86	1.26	2.63	0.32	−0.39	1.19
Pará	14	10	19	42	28	61	1.34	0.95	1.77	1.53	1.00	2.19	0.51	−0.26	1.41
Pernambuco	41	29	54	92	63	129	1.82	1.29	2.37	2.02	1.39	2.82	0.75	−0.05	1.79
Piaui	09	06	12	24	17	34	1.30	0.91	1.79	1.66	1.13	2.33	0.18	−0.56	1.01
Rio de Janeiro	136	89	177	266	180	359	2.72	1.78	3.59	2.54	1.72	3.43	0.58	−0.08	1.48
Rio Grande do Norte	11	08	15	29	20	39	1.55	1.10	2.04	1.72	1.20	2.38	0.58	−0.19	1.56
Rio Grande do Sul	82	56	108	163	105	229	2.45	1.63	3.24	2.25	1.45	3.16	0.93	0.11	2.15
Rondônia	03	02	04	09	06	13	1.39	0.97	1.95	1.42	0.94	2.11	0.26	−0.34	0.97
Roraima	01	00	01	02	02	03	1.78	1.25	2.33	1.82	1.27	2.48	0.53	−0.05	1.31
Santa Catarina	24	17	32	69	47	95	1.93	1.38	2.53	1.94	1.31	2.66	0.39	−0.40	1.35
Sergipe	06	05	08	18	12	25	1.54	1.12	1.98	1.88	1.25	2.60	0.69	−0.12	1.76
São Paulo	235	170	305	523	367	708	2.29	1.63	2.96	2.10	1.47	2.84	0.97	0.16	2.05
Tocantins	02	02	03	08	05	12	1.16	0.78	1.69	1.47	1.00	2.14	0.56	−0.24	1.68

^*^Age-standardized rate; U.I.: uncertainty interval.

In relation to breast cancer DALYs due to physical inactivity, 812,664 (95%U.I.: 585,946–1,048,865) DALYs were estimated in 1990 and 1,252,121 (95%U.I.: 908,348–1,612,391) in 2015 around the world. These values represented an age-standardized mortality rate of 40.32 (95%U.I.: 29.10–51.99) in 1990 and 34.48 (95%U.I.: 25.04–44.41) in 2015. In Brazil, 28,089 (95%U.I.: 20,840–35,448) DALYs were estimated in 1990 and 60,585 (95%U.I.: 44,213–77,331) in 2015, which represented an age-standardized mortality rate of 55.80 (95%U.I.: 41.48–70.51) in 1990 and 55.26 (95%U.I.: 40.39–70.58) in 2015. From 1990 to 2015, DALYs due to breast cancer attributable to physical inactivity remained stable around the world (−3.13%; 95%U.I.: −4.91–0.17), while Brazil had an increase (0.75%; 95%U.I.: 0.21–1.47). Table 3 shows the information on DALYs due to breast cancer attributable to physical inactivity for each Brazilian state.

**Table 3:** Number and age-standardized rate (per 100,000 inhabitants) of DALYs from breast cancer due to physical inactivity in women (≥25 years old) around the world, Brazil, and Brazilian states in 1990 and 2015.

	Breast cancer DALYs due to physical inactivity														
	1990			2015			1990			2015			Change (1990–2015)		
	DALYs	95% U.I.		DALYs	95% U.I.		Rate*	95% U.I.		Rate*	95% U.I.		%*	95% U.I.	
Global	812,664	585,949	1,048,865	1,252,121	908,348	1,612,391	40.32	29.10	51.99	34.48	25.04	44.41	−3.13	−4.91	0.17
Brazil	28,089	20,84	35,448	60,585	44,213	77,331	55.80	41.48	70.51	55.26	40.39	70.58	0.75	0.21	1.47
Acre	33	23	49	117	76	164	32.09	22.66	47.17	41.34	26.99	57.73	0.35	−0.33	1.17
Alagoas	318	223	424	739	517	1,003	42.60	30.00	57.10	47.72	33.38	64.73	0.39	−0.39	1.25
Amapá	17	11	28	82	50	135	24.91	16.31	42.57	32.75	19.98	53.83	0.64	−0.06	1.57
Amazonas	200	139	268	716	476	1,018	42.41	29.67	56.53	50.90	34.13	72.21	0.17	−0.54	1.04
Bahia	1,627	1,157	2,149	4,049	2,749	5,575	45.45	32.39	59.38	53.48	36.41	73.55	0.34	−0.40	1.15
Ceará	1,012	691	1,359	2,617	1,72	3,676	47.65	32.79	63.93	59.53	39.21	83.21	0.63	−0.11	1.68
Distrito Federal	269	189	356	761	515	1,061	55.35	38.87	72.16	49.15	33.56	68.37	0.51	−0.23	1.51
Espírito Santo	398	282	519	1,045	732	1,440	46.73	33.45	60.84	49.21	34.70	67.70	0.84	−0.02	1.97
Goiás	579	414	765	1,639	1,127	2,228	47.90	34.46	63.40	48.65	33.54	66.11	0.33	−0.49	1.36
Maranhão	474	318	664	1,267	807	1,832	34.72	23.19	49.27	45.12	29.10	64.94	0.43	−0.31	1.37
Mato Grosso	206	145	278	738	503	1,023	42.50	30.10	56.81	49.25	33.71	67.68	0.32	−0.61	1.16
Mato Grosso do Sul	254	187	337	664	458	923	47.75	35.05	62.81	49.68	34.34	68.49	0.08	−0.80	1.10
Minas Gerais	2,785	2,042	3,635	6,018	4,198	8,129	51.19	37.57	66.56	50.96	35.61	68.80	0.19	−0.62	1.14
Paraná	1,485	1,063	1,921	3,431	2,392	4,730	52.33	37.46	67.61	54.14	37.71	74.65	0.88	−0.01	1.95
Paraíba	476	338	624	1,082	734	1,533	42.93	30.45	56.17	52.66	35.83	74.35	0.38	−0.37	1.29
Pará	476	335	635	1,386	922	1,990	37.83	26.56	50.00	43.74	29.32	62.19	0.38	−0.46	1.29
Pernambuco	1,307	911	1,714	2,789	1,919	3,914	52.23	36.59	68.58	57.72	39.75	80.34	0.76	−0.10	1.85
Piaui	282	198	378	764	523	1,061	37.30	26.01	50.15	48.58	33.26	67.29	0.21	−0.62	1.15
Rio de Janeiro	4,282	2,825	5,569	7,488	5,108	10,129	76.46	50.23	99.70	71.33	48.57	96.71	0.61	−0.10	1.62
Rio Grande do Norte	342	242	451	842	579	1,159	43.06	30.64	56.67	48.29	33.30	66.62	0.55	−0.27	1.53
Rio Grande do Sul	2,545	1,744	3,361	4,368	2,873	6,188	67.82	46.03	89.59	60.66	39.86	86.01	0.72	−0.12	1.86
Rondônia	97	68	137	309	205	457	39.90	28.07	56.11	40.56	27.28	59.99	0.22	−0.49	1.00
Roraima	18	12	23	83	58	113	48.89	34.56	63.63	49.28	34.47	67.34	0.43	−0.16	1.21
Santa Catarina	790	565	1,047	2,037	1,379	2,831	53.44	38.15	70.63	53.98	36.65	74.72	0.26	−0.56	1.30
Sergipe	200	144	260	558	375	779	43.72	31.42	56.82	52.81	35.68	73.16	0.61	−0.17	1.69
São Paulo	7,535	5,472	9,768	14,727	10,331	19,718	63.46	45.91	82.34	56.56	39.71	76.02	0.88	0.07	1.97
Tocantins	76	50	108	259	177	372	32.71	21.70	47.44	41.58	28.64	59.26	0.50	−0.30	1.55

^*^Age-standardized rate; U.I.: uncertainty interval.

**Figure 1:**
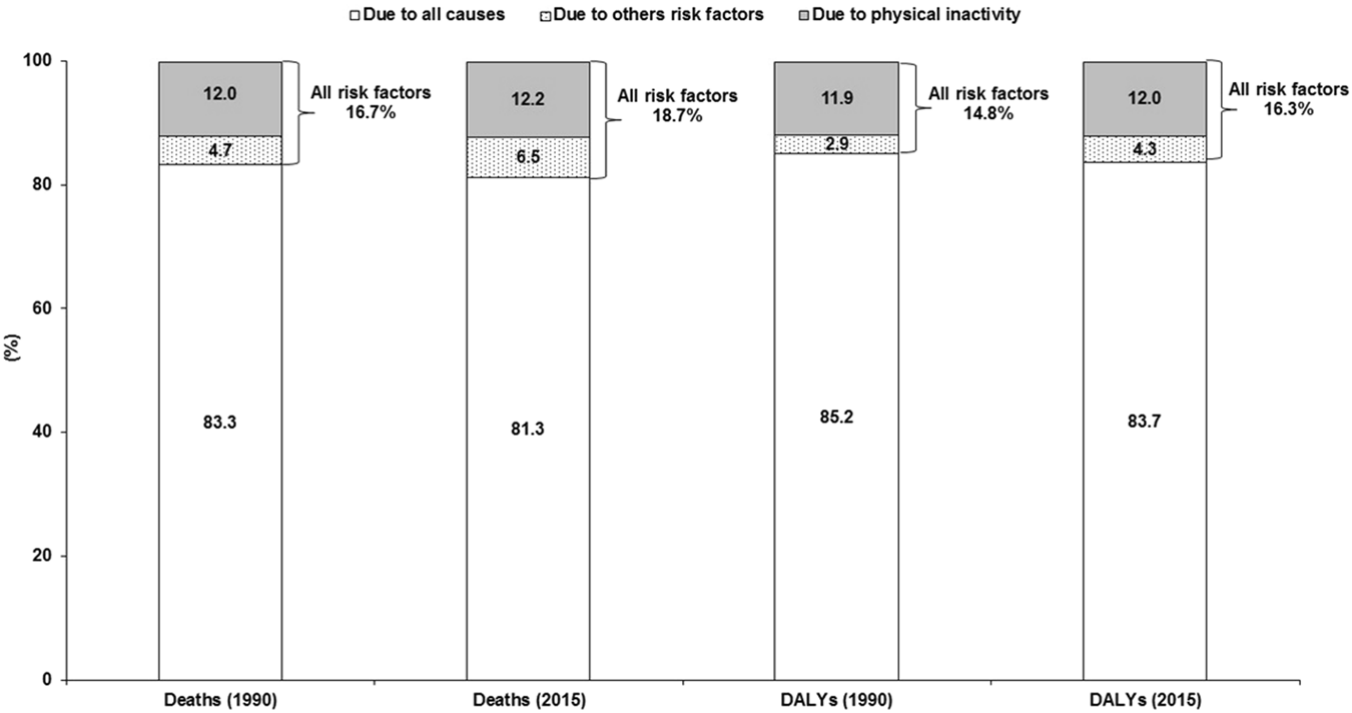
Percentage contribution of all causes, all risk factors and physical inactivity in mortality and DALYs by breast cancer in Brazilian women (≥25 years old). All risk factors = physical inactivity, alcohol use, high body-mass index, diet high in sugar-sweetened beverages.

Physical inactivity was responsible for 12.0% and 12.2% of all deaths due to breast cancer in Brazil in 1990 and 2015, respectively. The other risk factors (alcohol use, high body mass index, diet high in sugar-sweetened beverages) were responsible for 4.7% and 6.5% of all deaths due to breast cancer in Brazil in 1990 and 2015, respectively. Regarding DALYs due to breast cancer, physical inactivity was responsible for 11.9% and 12.0% of all DALYs in 1990 and 2015, respectively. The other risk factors (alcohol use, high body mass index, diet high in sugar-sweetened beverages) were responsible for 2.9% and 4.3% of all DALYs due to breast cancer in Brazil in 1990 and 2015, respectively (Fig. 1).

**Figure 2:**
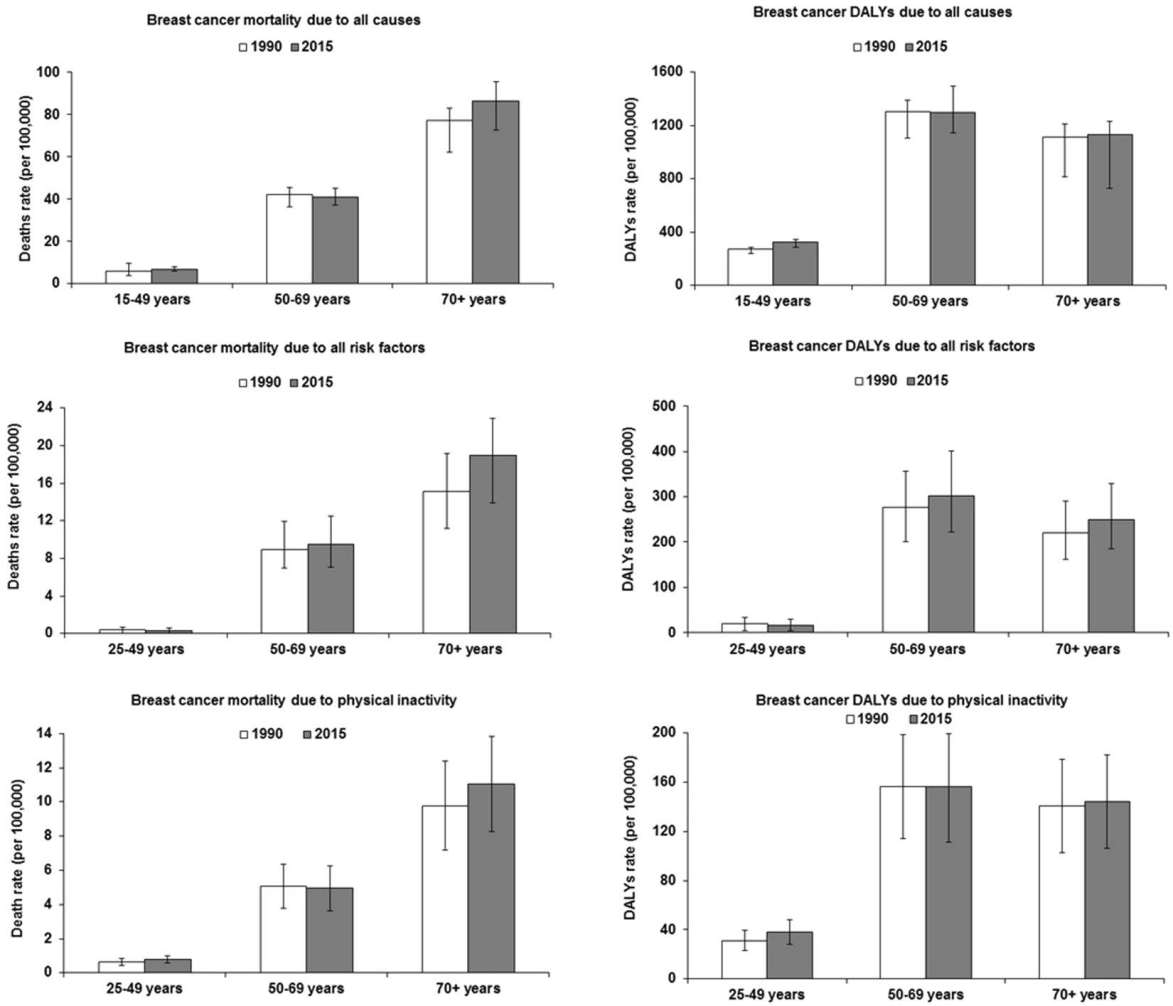
Rate and 95% uncertainty interval (per 100,000 inhabitants) of death and DALYs from breast cancer in women due to all causes, all risk factors, and physical inactivity in Brazil according to age. All risk factors = physical inactivity, alcohol use, high body-mass index, diet high in sugar-sweetened beverages.

In Brazil, mortality due to breast cancer attributable to all causes, all risk factors, and physical inactivity increased with increasing age in 1990 and 2015. DALYs due to breast cancer attributable to all causes, all risk factors, and physical inactivity were similar in the age groups 50–69 years and 70+ years (Fig. 2).

**Figure 3:**
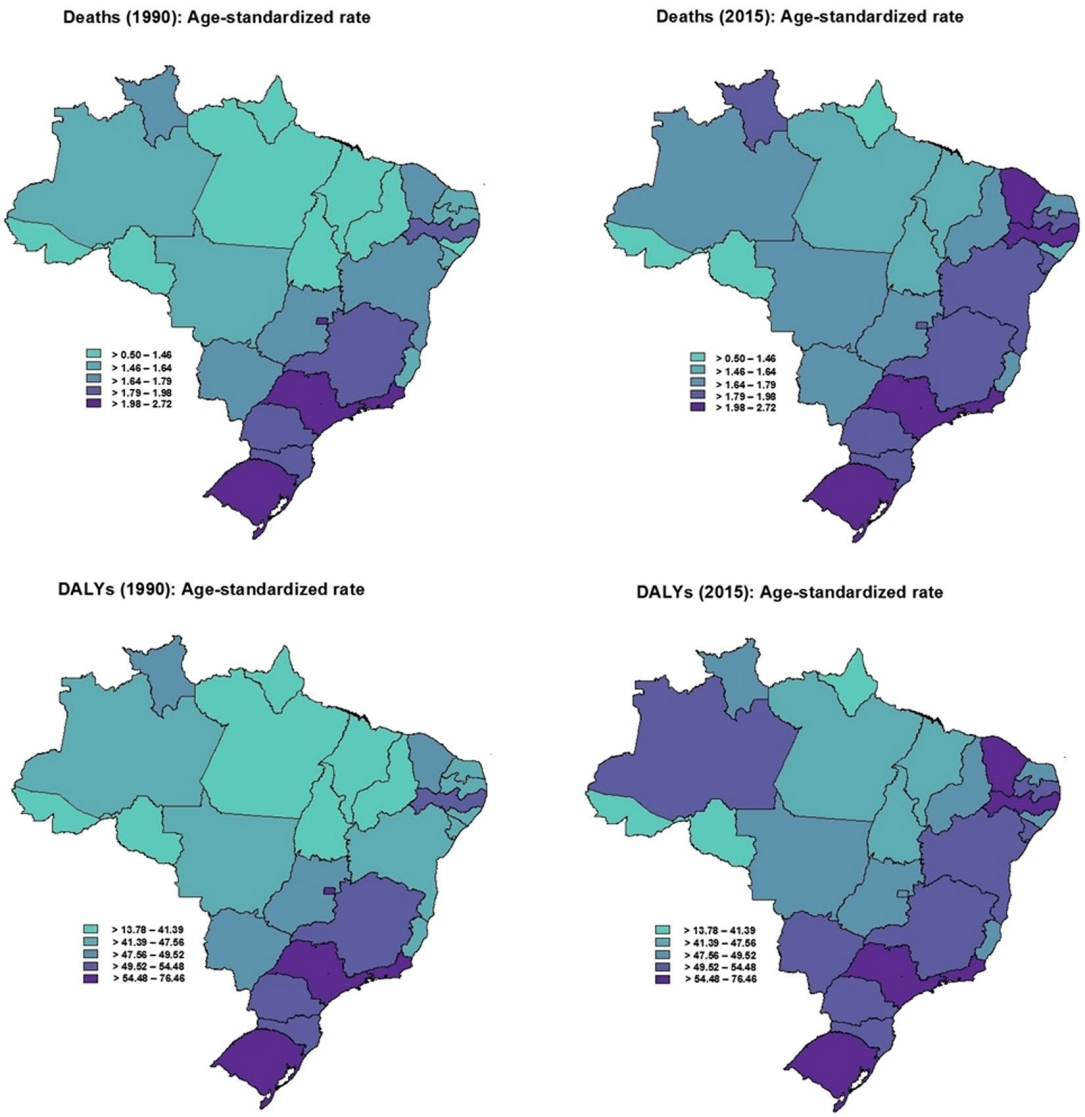
Age-standardized rate (per 100,000 inhabitants) of deaths and DALYs from breast cancer in women (≥25 years old) attributable to physical inactivity in the Brazilian states, 1990 and 2015.

**Figure 4:**
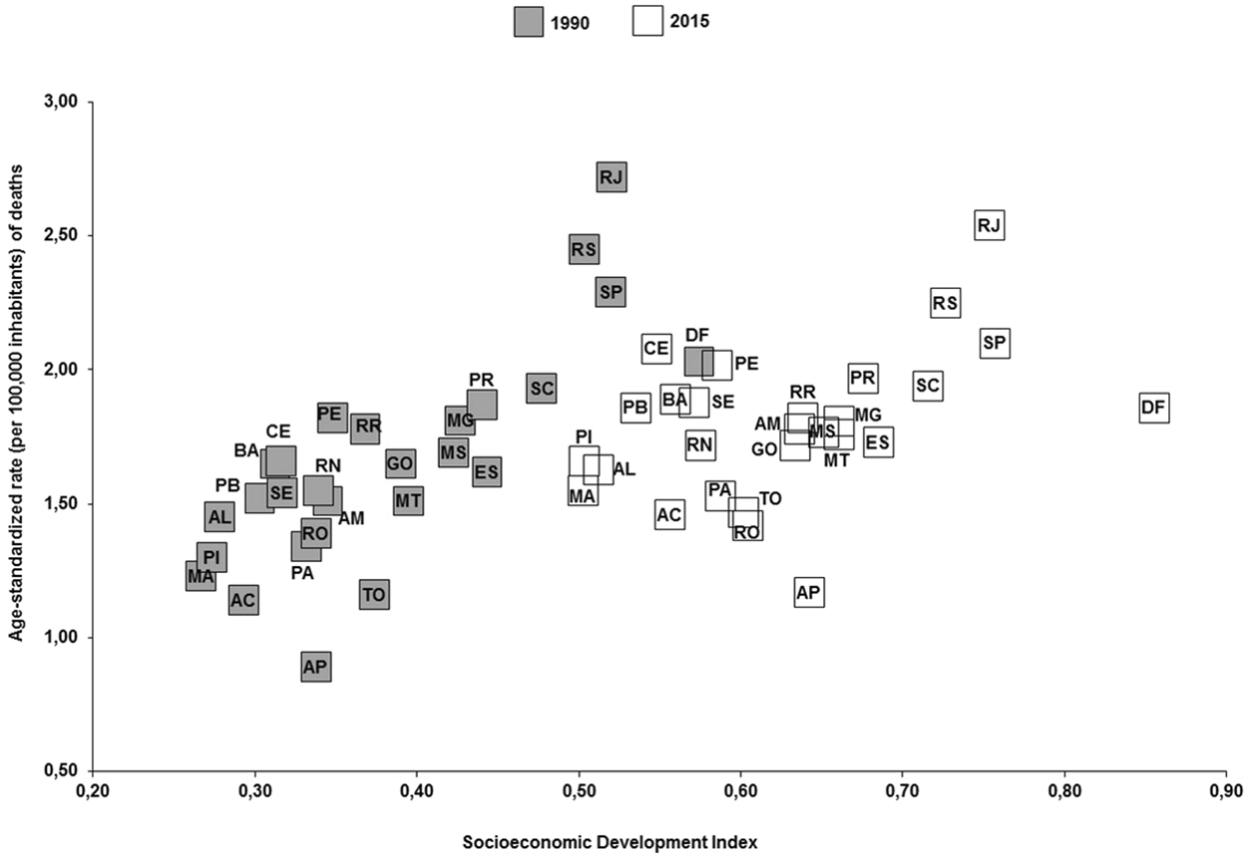
Relationship between age-standardized rate of deaths from breast cancer due to physical inactivity and Sociodemographic Index of the Brazilian states in 1990 and 2015. The Brazilian states with better Socioeconomic Development Index (SDI) showed higher age-standardized mortality rate (Rho: Spearman’s correlation coefficient; 1990: rho = 0.77, p < 0.01; 2015: rho = 0.47, p < 0.01). Brazilian states - AC: Acre; AL: Alagoas; AP: Amapá; AM: Amazonas; BA: Bahia; CE: Ceará; DF: Distrito Federal; ES: Espírito Santo; GO: Goiás; MA: Maranhão; MT: Mato Grosso; MS: Mato Grosso do Sul; MG: Minas Gerais; PA: Pará; PB: Paraíba; PR: Paraná; PE: Pernambuco; PI: Piauí; RR: Roraima; RO: Rondônia; RJ: Rio de Janeiro; RN: Rio Grande do Norte; RS: Rio Grande do Sul; SC: Santa Catarina; SP: São Paulo; SE: Sergipe; To: Tocantins.

**Figure 5:**
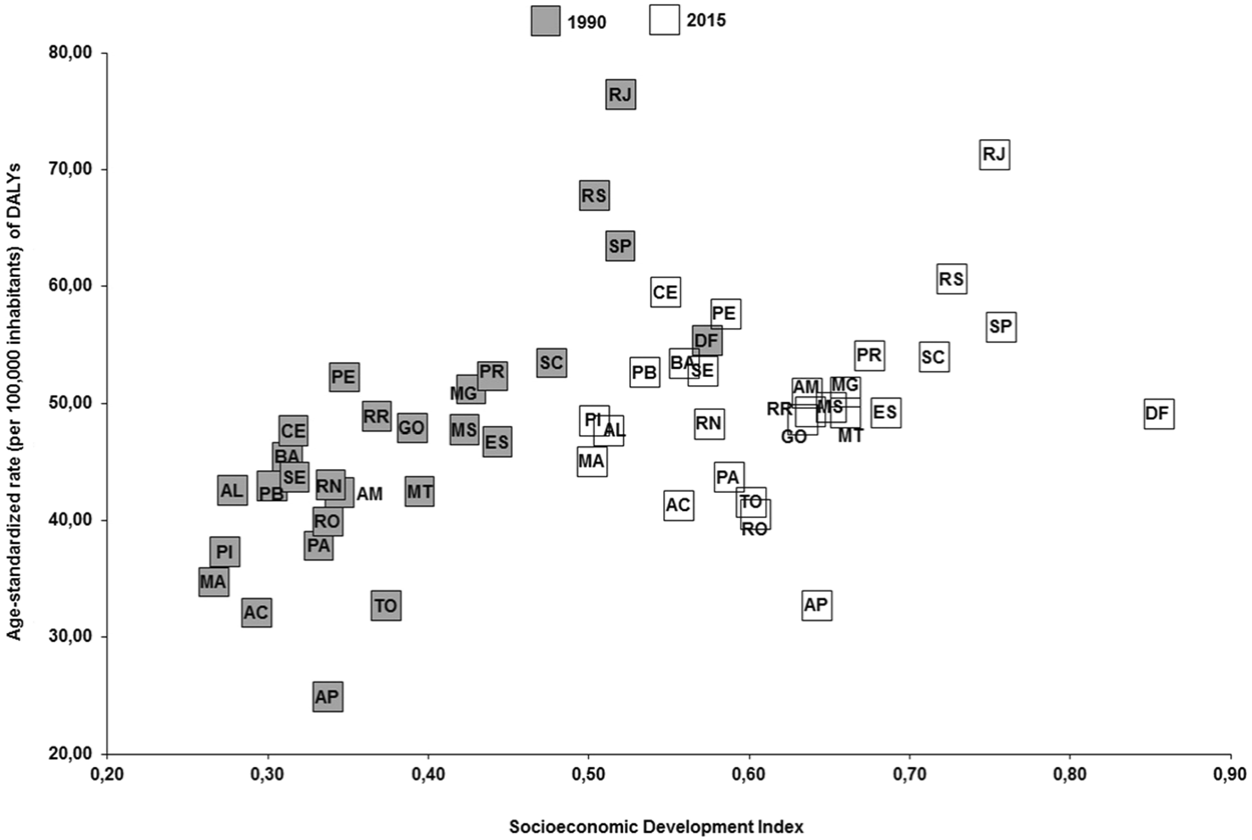
Relationship between age-standardized rate of DALYs from breast cancer due to physical inactivity and Socioeconomic Development Index of the Brazilian states in 1990 and 2015. The Brazilian states with better Socioeconomic Development Index showed higher age-standardized rate of DALYs (Rho: Spearman’s correlation coefficient; 1990: rho = 0.74, p < 0.01; 2015: rho = 0.42, p = 0.03). Brazilian states - AC: Acre; AL: Alagoas; AP: Amapá; AM: Amazonas; BA: Bahia; CE: Ceará; DF: Distrito Federal; ES: Espírito Santo; GO: Goiás; MA: Maranhão; MT: Mato Grosso; MS: Mato Grosso do Sul; MG: Minas Gerais; PA: Pará; PB: Paraíba; PR: Paraná; PE: Pernambuco; PI: Piauí; RR: Roraima; RO: Rondônia; RJ: Rio de Janeiro; RN: Rio Grande do Norte; RS: Rio Grande do Sul; SC: Santa Catarina; SP: São Paulo; SE: Sergipe; To: Tocantins.

The Brazilian states with better socioeconomic indicators showed higher age-standardized rates of deaths (1990: rho = 0.77, p < 0.01; 2015: rho = 0.47, p < 0.01) and DALYs (1990: rho = 0.74, p < 0.01; 2015: rho = 0.42, p = 0.03) due to breast cancer attributable to physical inactivity. The Brazilian states that showed higher age-standardized rates of deaths (per 100,000 inhabitants) due to breast cancer attributable to physical inactivity were Rio de Janeiro, southeastern Brazil (1990: 2.7, 95%U.I.: 1.8–3.6; 2015: 2.5, 95% U.I.: 1.7–3.4); Rio Grande do Sul, southern Brazil (1990: 2.5, 95%U.I.: 1.6–3.2; 2015: 2.3, 95% U.I.: 1.5–3.2); and São Paulo, southeastern Brazil (1990: 2.3, 95% U.I.: 1.6–3.0; 2015: 2.1, 95%U.I.: 1.5–2.8). The Brazilian states that showed higher age-standardized rates of DALYs (per 100,000 inhabitants) due to breast cancer attributable to physical inactivity were Rio de Janeiro, southeastern Brazil (1990: 76.5, 95%U.I.: 50.2–99.7; 2015: 71.3, 95%U.I.: 48.6–96.7); Rio Grande do Sul, southern Brazil (1990: 67.8, 95%U.I.: 46.0–89.6; 2015: 60.7, 95%U.I.: 39.9–86.0); and São Paulo, southeastern Brazil (1990: 63.5, 95%U.I.: 45.9–82.3; 2015: 56.6, 95%U.I.: 39.7–76.0) (Figs 3, 4 and 5).

## Discussion

Among the main findings of this study, it was found that over 25 years, estimates of deaths and DALYs due to breast cancer attributable to all causes were stable in Brazil and decreased around the world. A survey conducted in the Eastern Mediterranean Region analysed the trends of deaths and DALYs from 2005 to 2015 by different cancers and found that mortality and DALY rates due to breast cancer stabilized over a 10-year period^1^. In Denmark and Finland, a reduction in breast cancer mortality rates over 25 years (1985–1989 to 2009–2013) has been reported^15^, and in the U.S., there was a decline in breast cancer mortality rates from 1990 to 2009 in all ethnic groups^16^. From 1990 to 2015, public health policies were created in Brazil, which resulted in improvements in cancer screening and treatment services, such as the National Oncology Policy^17^. The improvement in health services may reflect in the larger diagnosis of cancer cases, which justifies the increased incidence in 2015. Additionally, this improvement in health services is perhaps reflected in the stability of the age-standardized rate of deaths and DALYs; however, the continued progress of these services is important for a significant decline^18,19^. According to the Brazilian Society of Radiotherapy, in 2013, 335 radiotherapy devices were needed to treat patients, but only 230 were available. As a result, approximately 90,000 patients stopped receiving radiotherapy in that year^19^. Thus, more effective actions for the prevention, screening and treatment of breast cancer are urgent in Brazil.

Among the risk factors for breast cancer, this study found that 12% of deaths and DALYs in Brazil were due to physical inactivity, and 4% to 6% were due to other risk factors (alcohol use, high body mass index, diet high in sugar-sweetened beverages). This percentage represented more than 3,000 deaths and more than 89,000 DALYs in Brazil due to breast cancer attributable to physical inactivity in the years analysed (1990 and 2015). A systematic review with meta-analysis^20^ analysed 22 prospective cohort studies that totalled 123,574 participants followed for a period ranging from 4.3 to 12.7 years and found that 5,462 subjects had breast cancer outcomes (e.g., breast cancer-related deaths or recurrences). Compared to those who reported low/no lifetime recreational pre-diagnosis physical activity, participants who reported high lifetime recreational pre-diagnosis physical activity levels had a significantly lower risk of breast cancer-related death. Significant risk reductions for breast cancer-related death was also demonstrated for more recent pre-diagnosis recreational physical activity, post-diagnosis physical activity, and meeting recommended post-diagnosis physical activity guidelines (e.g., ≥8 MET-h/wk)^20^. Another systematic review with meta-analysis^21^ reported results similar and added the information that the association between pre- or post-diagnosis physical activity and total mortality among breast cancer survivors did not differ according to BMI, menopausal status, or tumour oestrogen receptor status. These reviews concluded that regular physical activity is a protective factor for pre- and post-diagnosis breast cancer mortality and support the results of this study.

The biological mechanisms that explain the relationship between breast cancer and physical inactivity clarify why physical inactivity has contributed to deaths and DALYs. One of the factors that cause breast cancer is the excess of circulating sex hormones, especially oestrogen, which can lead to the formation of mutations and carcinogenesis by stimulating the production of free radicals exhibiting genotoxicity^22^. Physical activity decreases oestradiol and increases sex hormone-binding globulin, which is a steroid-binding plasma glycoprotein whose main function is to reduce the amount of oestradiol^23^. Another factor that can lead to breast cancer is hyperinsulinaemia, which promotes the synthesis and activity of insulin-like growth factor (IGF-1)^24^. Physical activity can reduce insulin levels and insulin resistance, thereby decreasing fasting glucose and total IGF-1^25^. Chronic inflammation is another factor that contributes to breast cancer development and progression through processes such as the polarization of immunosuppressive tumour-associated macrophages via cytokines and the subsequent production of tumour growth factors^26^. Physical exercise causes a reduction in the circulating amount of proinflammatory biomarkers and increases the circulating amount of anti-inflammatory substances^27^.

No studies that estimated the number of DALYs due to breast cancer attributable to physical inactivity were found. The studies found estimated the number of DALYs due to breast cancer attributable to all causes^1,9^, as this observation was also noted in the present study. Thus, this study adds to the growing body of evidence that healthy years of life are lost due to breast cancer, not only from all causes but specifically due to physical inactivity. Brazil had a stabilization of age-standardized rates of deaths and DALYs due to breast cancer attributable to physical inactivity over 25 years, and on the other hand, a decrease was observed around the world. The expansion and improvement of services for the screening and treatment of breast cancer and more effective actions to stimulate physical activity in the population may result in a decline in these rates in Brazil^18,19^. In the last 25 years, Brazil has implemented important public policies to encourage physical activity, such as the National Health Promotion Policy^28^, the creation of community physical activity programmes and the inclusion of physical activity in health monitoring and surveillance systems^28,29^. Additional actions are required, such as improving the built environment of the cities to enable physical activity^30^. Brazilian studies have shown that air pollution, few green areas and poor quality of urban infrastructure are still challenges^31,32^ that affect the engagement of the population in physical activities^30^. Another necessary action is to direct actions to encourage the practice of physical activity among children^33^. Data from the Active Healthy Kids Global Alliance have demonstrated that only half of children in Brazil meet physical activity recommendations and that there are few government incentives to promote physical activity in this age group^33^.

We found an increase in mortality and DALYs due to breast cancer attributable to physical inactivity with advancing age. The literature has shown that physical inactivity increases with age^34^, and this finding may justify our results. This study found that age-standardized rates of deaths and DALYs due to breast cancer attributable to physical inactivity were higher in Brazilian states with better socioeconomic conditions. According to the World Health Organization^35^, the increase in income and improvements in living standards in developing countries (which are observed more frequently in states with better socioeconomic conditions) have been accompanied by an increase in the incidence of breast cancer in women. This finding may be due to factors such as longer life, greater exposure to risk factors, higher fat intake, lower levels of physical activity, obesity and lower pregnancy rates than those of women who live in cities with worse economic conditions^35^. Another factor that may explain the high mortality and DALYs due to breast cancer attributable to physical inactivity in Brazilian states with better socioeconomic conditions is the fact that in these states, health services are better structured and serve more people than in states with worse socioeconomic conditions^18^. Serving more people may result in more diagnoses of breast cancer according to Brazilian Oncology Observatory^18^.

The number of breast cancer survivors in Brazil was not investigated in the present study; however, this number may also have increased during the 25 years, since there have been improvements in health services^18,19^. For this particular population, regular physical activity has physical and psychological benefits^20,21^. In addition, it can prevent recurrence of the disease^20,21^. Thus, physical activity should be prioritized at any stage of breast cancer.

The findings from this study can be useful for improving public health in Brazil because we found a large number of deaths and DALYs due to breast cancer attributable to modifiable risk factors. This information reinforces the need to act in the prevention of breast cancer not only through mammography screening but also through a healthy lifestyle. Adopting a healthy lifestyle has a lower cost to public health than the future treatment of the disease^2^. Thus, the present study reinforces the need for public policies focused on primary health care^5^. On the other hand, it can be inferred from this study that modifiable risk factors can help in tertiary prevention^7,8^, since different research has shown that adopting a healthy lifestyle should be a therapeutic adjunct for breast cancer patients^20,21^. This study did not stratify breast cancer cases according to the staging of the disease, but the numbers of deaths and DALYs attributable to physical inactivity and other risk factors provide benchmark for public health in Brazil in its prioritization of healthy lifestyles for women who both have and have not received a diagnosis of breast cancer.

This study has important limitations. First, this study did not stratify by the type of breast cancer to identify which type has the greatest relation with physical inactivity^36^. Second, this study did not exclude genetic cases of breast cancer mutations and did not stratify breast cancer according to the different types of treatment (e.g., chemotherapy with anthracyclines, radiotherapy, or other) to identify which has the greatest relation with physical inactivity^37^. Third, our study does not include all risk factors for breast cancer, such as the dosage of hormone replacement therapy and other pharmaceutical factors considered to be risks for this disease. Fourth, the present study only includes physical activity measured by questionnaires that are considered subjective measures of physical activity and are associated with measurement bias^38^. Fifth, the non-stratification of physical activity by domains is another limitation. Sixth, the use of a simulation model may be considered another limitation, although this model has evidence of validity^39^.

The strength of this study was the estimation of mortality and DALYs by physical inactivity throughout Brazil. From this information, it was possible to estimate mortality rates and DALYs due to breast cancer attributable to physical inactivity. This is the first study that used data from all states to make these estimates, which increases the accuracy and resolution of the information. Thus far, it has been known that physical inactivity was a risk factor for breast cancer, but it had not been estimated the degree to which it was related to morbidity (DALYs) and mortality, especially across two time periods (1990 and 2015).

It could be concluded that physical inactivity has contributed to a substantial number of deaths and DALYs due to breast cancer in Brazil and around the world. Physical inactivity was responsible for more deaths and DALYs due to breast cancer than other modifiable risk factors. Estimates of mortality and morbidity from breast cancer as a result of physical inactivity in Brazil increased between 1990 and 2015 compared to global estimates. Brazilian states with better socioeconomic indicators had higher mortality and morbidity rates from breast cancer due to physical inactivity. These findings support the promotion of physical activity in the Brazilian female population to help prevent and manage breast cancer.

## Methods

### Study overview

The Global Burden of Disease Study (GBD) 2015 includes an annual assessment covering 195 countries and territories from 1990 to 2015. It covers 310 diseases and injuries, 2,619 sequelae and 79 risk factors by age and sex. Detailed descriptions of the methodology and approach of the GBD 2015 have been published elsewhere^13,14,40^.

Data from this study included only information on the female population. For data on the association between breast cancer and physical activity and between breast cancer and other risk factors, the information corresponds to women aged ≥25 years.

### Breast cancer estimates

All neoplasms, as defined by the 10^th^ revision of the International Statistical Classification of Diseases (ICD-10) to one of the 29 GBD cancer groups, were mapped. ICD-10 codes for the incidence, morbidity and mortality due to breast cancer were C50-C50.929, D05-D05.92, D24-D24.9, D48.6-D48.62, D49.3, and N60-N60.99^1,9^.

Input data for cancer mortality estimates came from the vital statistics mortality data and the cancer registry incidence data. The latter were transformed into mortality estimates using separately modelled mortality-to-incidence ratios (MIR)^1,9^. Crude data were processed to make them comparable and to account for “garbage codes”, which are codes assigned to causes that are not usable from the perspective of public health^40^. These causes were redistributed to the most likely underlying cause of death based on the regression model^1^. Through the use of a Cause of Death Ensemble modelling (CODEm) approach with cause-specific covariates, mortality estimates for each individual cause were computed^41^. These estimates were scaled to fit into an independently modelled all-cause mortality estimate using the CoDCorrect algorithm^39^.

Cancer survival was calculated using a MIR-based scaling factor. A 10-year prevalence of each cancer and each incidence cohort was calculated using these cancer survival estimates. The total prevalence was divided into four sequelae with variable disability weights: (1) diagnosis and treatment, (2) remission, (3) metastasis, and (4) terminal phase. A constant duration for sequelae (1), (3), and (4) was assumed. Sequela duration (2) was the remaining prevalence after subtracting the fixed sequelae duration. Years of life lost (YLLs) were computed by multiplying deaths by the normative standard life expectancy at each age of death^39^. For each sequela, years lost due to disability (YLDs) were calculated by multiplying the prevalence of each sequela by its disability weight. Finally, DALYs were calculated by summing premature death (YLLs) and YLDs. More information about these estimates can be found elsewhere^39^.

### Physical inactivity estimate

Surveys of the general adult population performed using random sampling procedures that assessed self-reported physical activity in all life domains (leisure/recreation, work, household and transport) were included. Due to the absence of a consistent relationship at the individual level between the amount of physical activity performed in each domain and total activity, it was not possible to use studies that included only recreational/leisure activities^12^.

For the global estimates, data were primarily derived from two standardized questionnaires, the Global Physical Activity Questionnaire (GPAQ) and the International Physical Activity Questionnaire (IPAQ), although any other survey instrument that asked about intensity, frequency and duration of physical activities performed across all activity domains was included^12^.

In the case of Brazil, surveys such as the telephone-based Surveillance of Risk and Protective Factors for Chronic Diseases, the Brazil World Health Survey, and the International Prevalence Study on Physical Activity were also consulted^13,14^. More details can be found at http://ghdx.healthdata.org/gbd-2015/data-input-sources and in a previous publication^12^.

To standardize all estimates of physical inactivity in Brazil and around the world, data from the population aged 25 years or more were considered. Reported physical activity was accumulated for durations of at least ten consecutive minutes across all life domains. Physical activity frequency, duration and intensity were used to calculate the total metabolic equivalent (MET) minutes per week^12^. Estimates were made for subjects classified as physically inactive (<600 METS-min/week)^12^.

### Others risk factor estimates (alcohol use, high body-mass index, diet high in sugar-sweetened beverages)

In addition to physical inactivity, the GBD study defined other modifiable risk factors for breast cancer. This definition took into account the quality of the information, the evidence, and the quality of the prediction models^12^.

For alcohol use, the theoretical minimum-risk exposure level (TMREL) was assumed to be no alcohol use (e.g., 0 g/day)^12^. Thus, it was considered a risk if there were some alcohol consumption/day. To generate estimates of alcohol consumption in grams per day, data from population surveys were used in combination with estimates of per capita consumption from the Food and Agriculture Organization^42^ and the Global Information System on Alcohol and Health database^43^. Per capita consumption is an aggregate measure of recorded, unrecorded, and tourist per capita consumption of alcohol (UNWTO database^44,45^) derived from sales, production, and other economic statistics. While population-based surveys provide accurate estimates of the prevalence of lifetime abstainers, former drinkers and current drinkers, they typically underestimate real alcohol consumption levels^12^. As a result, the per capita consumption figures across all ages and both sexes from the FAO and GISAH are considered to be a better estimate of overall volume of consumption^12^. Per capita consumption, however, does not provide the age- and sex-specific consumption estimates needed to compute alcohol-attributable burden of disease. Therefore, we use the age-sex pattern of consumption among drinkers modelled from the population survey data and the overall volume of consumption from the FAO and GISAH to determine the total amount of alcohol consumed by the country and states.

The TMREL of BMI was determined based on the BMI level that was associated with the lowest risk of all-cause mortality in prospective cohort studies^43^. In this study, the TMREL of BMI was 20–25 kg/m². Thus, a BMI > 25 kg/m² was considered a risk. We used data from multi-country survey programmes, national surveys, and longitudinal studies that were available in the Global Health Data Exchange (GHDx) and provided either self-report or measured data on height and weight. A complete description of the data-seeking and update process for the GHDx is provided elsewhere^12^. In addition, a systematic review of national and subnational studies was conducted for studies published until 31 December 2015^12^. We adjusted self-reported data for overweight prevalence, obesity prevalence, and mean BMI using hierarchical mixed-effects regression models, fit using maximum likelihood separately by sex.

Exposure to a diet high in sugar-sweetened beverages was defined as an average daily consumption of greater than 2.5 grams per day of beverages with ≥50 kcal per 226.8-gram serving, including carbonated beverages, sodas, energy drinks, and fruit drinks, but excluding 100% fruit and vegetable juices. In this study, the TMREL of diets high in sugar-sweetened beverages was 0–5 grams/day. Thus, consumption greater than 5 grams/day was considered a risk^12^. We used dietary data from multiple sources, including nationally and sub-nationally representative nutrition surveys, household budget surveys, and the United Nations FAO food balance sheets and supply utilization accounts. All dietary data were standardized to 2000 kcal/day. For each dietary factor, we estimated the global age pattern of consumption based on nutrition surveys (e.g., 24-hour diet recall) and applied that age pattern to the FAO data.

Further details about the modelling used for the risk factors were previously published^12^. Brazil’s surveys for all these risk factors are available at http://ghdx.healthdata.org/gbd-2015/data-input-sources.

### Analytical methods

The contribution of all causes, all risk factors and physical inactivity to mortality and DALYs due to breast cancer was estimated using a comparative risk assessment approach in which observed health outcomes are compared to those that would have been observed with a counterfactual set of exposures where no one was exposed^12^. For this approach, we used the simulation model by the CODEm to estimate indicators by age, sex, country, state, year, and cause, that is, an analytical tool that tests several possible statistical models of causes of death and creates a combined set of models that offers the best predictive performance. The DisMod-MR 2.1 software (World Health Organization©, Geneva, Switzerland), a meta-regression tool, was used for the derivation of simultaneous estimates of incidence, prevalence, remission, disability, and mortality attributable to risk factors, such as physical inactivity^12,39^. In this study, the results of these models were used to proportionally distribute deaths from breast cancer from all causes, all risk factors, and physical inactivity^39^. Modelling details can be found in the literature^12^.

Incident breast cancer cases, absolute number of deaths, mortality rates and DALYs (per 100,000 inhabitants—crude and age-standardized) were used as metrics. The sum of DALYs across the population, or the burden of the disease, can be considered a measurement of the gap between current health status and the ideal health situation^46^.

In the GBD study, 95% uncertainty intervals (95%U.I.) were calculated to provide information on the variability of estimates resulting from errors due to the sampling process and non-sample errors due to adjustments of data sources and modelling^39^.

The GBD 2015 created the Socioeconomic Development Index (SDI)^39^ for all evaluated locations by calculating per capita income, formal education at 15 years of age and fertility rate. This index was used to compare the metrics used among Brazilian states. For this analysis, the Spearman correlation coefficient was applied.

The datasets generated during and/or analysed during the current study are available from the corresponding author upon reasonable request. All data generated or analysed during this study are included in this published article (and its supplementary information files).

## Electronic supplementary material

Supplementary Information
