# Supplementary material for: Mortality and years of life lost due to breast cancer attributable to physical inactivity in the Brazilian female population (1990–2015)
Source: Sci Rep. 2018 Jul 24;8:11141. doi: 10.1038/s41598-018-29467-7 (PMC6057969; doi:10.1038/s41598-018-29467-7)
Supplement: Supplementary file 1 — Supplementary Information [file 41598_2018_29467_MOESM1_ESM.pdf]

Supplementary

Mortality and years of life lost due to breast cancer attributable to physical inactivity in the Brazilian female population (1990-2015)

Diego Augusto Santos Silva[1]*, Mark Stephen Tremblay[2], Maria de Fatima Marinho de Souza[3], Maximiliano Ribeiro Guerra[4], Meghan Mooney[5], Mohsen Naghavi[5], Deborah Carvalho Malta[6]

1. Federal University of Santa Catarina, Research Center in Kinanthropometry and Human Performance. Florianopolis, SC, 88040-900, Brazil.
2. Children's Hospital of Eastern Ontario Research Institute, Ottawa, ON, ONK1H5B2, Canada.
3. Ministry of Health, Department of Surveillance of Noncommunicable Diseases, and Injuries, and Health Promotion, Brasília, DF, 70058-900, Brazil.
4. Federal University of Juiz de Fora, Post-graduate Program in Public Health, Juiz de Fora, MG, 36036-330, Brazil.
5. Institute for Health Metrics and Evaluation. Seattle, WA 98121, United Sates.
6. Federal University of Minas Gerais, Department of Maternal and Child Nursing and Public Health, School of Nursing, Belo Horizonte, MG, 31270-901, Brazil.

*Corresponding Author: diegoaugustoss@yahoo.com.br

Supplementary Table S1. Number and age-standardized rate (per 100,000 inhabitants) of deaths from breast cancer due to all causes in women (≥ 15 years old) around the world, Brazil, and Brazilian states in 1990 and 2015.

	Breast cancer mortality due to all causes														
	**1990**			**2015**			**1990**			**2015**			**Change (1990-2015)**		
	**Deaths**	**95% U.I.**		**Deaths**	**95% U.I.**		**Rate***	**95% U.I.**		**Rate***	**95% U.I.**		**%***	**95% U.I.**	
Global	324,867	312,956	347,331	523,487	492,250	543,275	16.61	16.02	17.73	14.58	13.72	15.13	-0.12	-0.18	-0.08
Brazil	7,264	6,185	7,694	16,964	14,880	18,402	16.35	13.81	17.30	16.26	14.22	17.64	-0.01	-0.11	0.10
Acre	08	06	11	28	21	38	9.04	7.50	13.21	11.49	8.83	15.07	0.27	-0.05	0.68
Alagoas	77	65	93	184	142	234	11.51	9.78	13.90	12.93	9.99	16.51	0.12	-0.15	0.47
Amapá	04	03	07	19	13	32	7.07	5.51	12.14	9.25	6.42	14.92	0.31	-0.10	0.90
Amazonas	46	39	56	168	128	218	11.82	10.01	14.31	13.97	10.82	17.90	0.18	-0.15	0.59
Bahia	426	358	494	1,083	845	1,345	13.26	11.15	15.39	15.15	11.84	18.73	0.14	-0.13	0.50
Ceará	261	206	306	702	527	880	13.51	10.90	15.72	16.75	12.64	20.84	0.24	-0.06	0.61
Distrito Federal	64	55	73	221	171	278	16.80	14.15	19.24	15.38	11.97	19.38	-0.08	-0.30	0.18
Espírito Santo	101	89	116	287	227	356	13.52	11.92	15.58	14.28	11.34	17.70	0.06	-0.20	0.34
Goiás	134	117	160	414	336	507	13.19	11.49	15.79	13.63	11.11	16.71	0.03	-0.18	0.29
Maranhão	120	89	155	326	233	433	9.96	7.33	12.76	12.52	9.00	16.55	0.26	-0.13	0.86
Mato Grosso	46	39	56	179	137	225	11.89	9.93	14.52	13.80	10.77	17.04	0.16	-0.15	0.54
Mato Grosso do Sul	63	54	73	181	139	228	14.01	12.19	16.17	14.69	11.44	18.31	0.05	-0.20	0.35
Minas Gerais	728	637	830	1,717	1,400	2,098	15.06	13.16	17.14	15.02	12.29	18.31	0.00	-0.22	0.28
Paraná	377	328	430	972	770	1,211	15.53	13.41	17.76	16.24	12.91	20.16	0.05	-0.19	0.34
Paraíba	126	106	147	293	221	378	12.20	10.37	14.16	14.82	11.19	19.08	0.22	-0.11	0.65
Pará	117	98	142	344	254	459	10.82	9.05	13.06	12.33	9.23	16.29	0.14	-0.18	0.55
Pernambuco	342	290	398	754	584	960	15.04	12.77	17.44	16.53	12.85	21.01	0.10	-0.16	0.44
Piaui	70	56	87	195	148	248	10.34	8.32	13.12	13.18	10.10	16.77	0.27	-0.07	0.76
Rio de Janeiro	1,105	755	1,246	2,140	1,542	2,565	22.02	14.88	24.90	20.44	14.73	24.51	-0.07	-0.24	0.15
Rio Grande do Norte	92	78	106	233	184	290	12.64	10.65	14.60	13.95	11.07	17.26	0.10	-0.14	0.39
Rio Grande do Sul	691	499	795	1,346	980	1,748	20.47	14.64	23.62	18.58	13.53	24.15	-0.09	-0.31	0.22
Rondônia	20	17	28	70	53	96	10.69	8.90	14.45	10.83	8.23	15.03	0.01	-0.24	0.33
Roraima	04	03	05	19	15	24	13.86	11.58	15.98	14.13	11.33	17.29	0.02	-0.22	0.27
Santa Catarina	202	172	231	566	441	725	15.89	13.45	18.17	15.91	12.43	20.29	0.00	-0.25	0.32
Sergipe	53	45	62	148	112	188	12.68	10.79	14.74	15.39	11.73	19.37	0.21	-0.08	0.58
São Paulo	1,968	1,555	2,199	4,303	3,487	5,168	18.96	14.78	21.23	17.18	13.93	20.61	-0.09	-0.28	0.12
Tocantins	19	14	26	66	50	90	9.47	7.03	13.20	11.92	9.06	16.18	0.26	-0.13	0.85

*Age-standardized rate; U.I.: uncertainty interval.

Supplementary Table S2. Number and age-standardized rate (per 100,000 inhabitants) of DALYs from breast cancer due to all causes in women (≥ 15 years old) around the world, Brazil, and Brazilian states in 1990 and 2015.

	**Breast cancer DALYs due to all causes**														
	**1990**			**2015**			**1990**			**2015**			**Change (1990-2015)**		
	**DALYs**	**95% U.I.**		**DALYs**	**95% U.I.**		**Rate***	**95% U.I.**		**Rate***	**95% U.I.**		**%***	**95% U.I.**	
Global	9,578,973	9,067,559	10,395,449	15,137,828	14,156,028	15,936,286	469.16	444.78	508.02	414.23	387.26	436.26	-0.12	-0.18	-0.06
Brazil	236,482	205,941	251,674	503,463	443,581	548,345	465.87	401.66	495.34	458.03	402.36	499.60	-0.02	-0.12	0.09
Acre	266	219	390	942	724	1,247	256.73	211.22	376.25	329.49	254.99	429.98	0.28	-0.04	0.69
Alagoas	2,579	2,184	3,107	5,949	4,61	7,578	342.94	291.97	412.12	382.75	296.76	484.50	0.12	-0.16	0.46
Amapá	135,7	104,42	226,07	669	459	1,074	201.36	155.74	337.17	263.32	182.32	416.37	0.31	-0.10	0.88
Amazonas	1,615	1,351	1,940	5,741	4,391	7,441	337.25	284.11	404.74	404.02	314.78	519.88	0.20	-0.13	0.64
Bahia	13,400	11,140	15,609	33,065	25,585	41,065	371.10	310.81	430.14	435.42	338.36	539.94	0.17	-0.11	0.53
Ceará	8,406	6,461	9,973	21,507	15,722	26,909	393.04	304.79	464.06	487.93	358.05	608.09	0.24	-0.04	0.61
Distrito Federal	2,312	2,002	2,661	6,437	4,887	8,118	467.86	401.06	533.61	413.59	314.52	519.09	-0.12	-0.34	0.16
Espírito Santo	3,414	2,991	3,943	8,833	7,039	10,891	396.78	349.22	456.47	414.49	330.22	511.38	0.04	-0.21	0.32
Goiás	4,737	4,088	5,616	13,296	10,814	16,324	387.81	337.23	460.08	392.58	321.44	479.40	0.01	-0.21	0.27
Maranhão	3,939	2,957	5,017	10,44	7,509	13,921	285.98	213.93	364.47	369.97	267.38	488.88	0.29	-0.11	0.88
Mato Grosso	1,669	1,390	2,035	5,916	4,515	7,439	339.08	284.39	412.16	392.03	302.68	491.67	0.16	-0.16	0.52
Mato Grosso do Sul	2,178	1,872	2,534	5,654	4,338	7,13	404.09	350.70	467.57	420.13	324.65	526.66	0.04	-0.22	0.35
Minas Gerais	23,756	20,994	27,380	50,961	41,427	62,086	433.08	381.68	494.59	430.63	350.64	525.13	-0.01	-0.22	0.27
Paraná	12,729	11,031	14,471	28,901	23,093	35,908	443.38	385.45	501.34	454.58	363.01	562.61	0.03	-0.21	0.30
Paraíba	3,899	3,289	4,541	8,79	6,623	11,184	349.66	295.40	406.02	427.07	322.33	542.48	0.22	-0.10	0.65
Pará	3,960	3,279	4,804	11,442	8,523	15,086	310.72	259.82	374.28	357.95	269.25	469.82	0.15	-0.16	0.58
Pernambuco	11,045	9,296	12,945	23,272	17,979	29,549	438.06	368.99	509.52	480.15	372.64	605.75	0.10	-0.17	0.43
Piaui	2,303	1,852	2,815	6,183	4,741	7,777	301.22	242.68	369.81	391.76	302.08	490.72	0.30	-0.04	0.76
Rio de Janeiro	35,393	24,788	40,041	61,192	44,364	73,452	628.44	436.38	708.47	583.05	421.95	699.97	-0.07	-0.26	0.15
Rio Grande do Norte	2,850	2,383	3,296	6,957	5,452	8,646	356.56	300.18	412.20	397.61	313.65	493.24	0.12	-0.13	0.43
Rio Grande do Sul	21,821	16,103	25,037	36,883	27,694	48,167	577.52	422.07	663.30	512.64	383.98	667.04	-0.11	-0.33	0.19
Rondônia	760	623	1,022	2,401	1,818	3,319	309.54	255.91	417.31	313.79	240.92	432.02	0.01	-0.24	0.32
Roraima	140	116	162	656	522	811	385.64	322.60	442.71	387.13	311.00	473.47	0.00	-0.22	0.25
Santa Catarina	6,704	5,753	7,612	17,099	13,409	21,841	448.46	384.64	507.96	451.64	354.20	574.57	0.01	-0.25	0.32
Sergipe	1,704	1,43	1,975	4,698	3,507	5,892	367.90	310.76	427.14	441.59	334.46	551.48	0.20	-0.09	0.58
São Paulo	64,122	51,802	71,503	123,409	100,122	147,422	535.40	426.35	596.85	473.05	384.54	564.06	-0.12	-0.30	0.09
Tocantins	634	469	862	2,156	1,616	2,914	271.67	202.80	369.53	343.79	259.23	462.56	0.27	-0.12	0.86

*Age-standardized rate; U.I.: uncertainty interval.

Supplementary Table S3. Number and age-standardized rate (per 100,000 inhabitants) of deaths from breast cancer due to all risk factors (physical inactivity, alcohol use, high body-mass index, diet high in sugar-sweetened beverages) in women (≥ 25 years old) around the world, Brazil, and Brazilian states in 1990 and 2015.

	**Breast cancer mortality due to all risk factors**														
	**1990**			**2015**			**1990**			**2015**			**Change (1990-2015)**		
	**Deaths**	**95% U.I.**		**Deaths**	**95% U.I.**		**Rate***	**95% U.I.**		**Rate***	**95% U.I.**		**%***	**95% U.I.**	
Global	63,265	52,177	73,225	103,829	84,505	123,222	3.28	2.70	3.79	2.91	2.37	3.46	-0.11	-0.17	-0.05
Brazil	1,212	922	1,527	3,166	2,359	4,149	2.88	2.19	3.66	3.10	2.30	4.05	0.07	-0.05	0.20
Acre	01	01	02	05	03	07	1.59	1.12	2.37	2.15	1.44	3.07	0.35	0.00	0.82
Alagoas	13	09	17	32	22	45	1.99	1.49	2.68	2.39	1.66	3.31	0.20	-0.10	0.58
Amapá	01	00	01	03	02	05	1.25	0.82	2.27	1.77	1.10	2.83	0.41	-0.03	1.04
Amazonas	07	05	10	29	19	42	2.13	1.53	2.89	2.67	1.80	3.89	0.26	-0.09	0.72
Bahia	72	54	95	197	138	273	2.36	1.75	3.12	2.86	2.01	3.95	0.21	-0.09	0.59
Ceará	43	31	57	126	85	175	2.35	1.66	3.11	3.11	2.11	4.32	0.32	0.00	0.72
Distrito Federal	10	07	13	40	27	57	2.97	2.15	4.02	3.00	2.03	4.17	0.01	-0.25	0.31
Espírito Santo	16	12	21	52	36	71	2.31	1.69	3.03	2.64	1.84	3.62	0.14	-0.15	0.47
Goiás	22	16	28	74	52	101	2.32	1.73	3.06	2.58	1.82	3.55	0.11	-0.13	0.41
Maranhão	19	13	27	55	35	80	1.69	1.16	2.37	2.25	1.44	3.25	0.33	-0.08	0.97
Mato Grosso	07	05	10	32	22	45	2.13	1.54	2.85	2.66	1.81	3.67	0.25	-0.08	0.65
Mato Grosso do Sul	10	07	13	32	22	46	2.41	1.77	3.11	2.75	1.93	3.88	0.14	-0.16	0.47
Minas Gerais	120	90	153	317	224	423	2.61	1.96	3.36	2.80	1.97	3.72	0.07	-0.17	0.40
Paraná	60	45	78	180	125	244	2.69	2.00	3.51	3.06	2.12	4.15	0.14	-0.13	0.46
Paraíba	21	16	28	53	36	74	2.12	1.56	2.78	2.77	1.86	3.86	0.31	-0.04	0.76
Pará	19	14	25	59	39	86	1.88	1.38	2.50	2.29	1.53	3.33	0.22	-0.12	0.69
Pernambuco	56	42	73	137	94	195	2.57	1.91	3.34	3.11	2.14	4.35	0.21	-0.08	0.58
Piaui	12	08	16	35	25	49	1.81	1.29	2.49	2.48	1.72	3.40	0.37	-0.01	0.87
Rio de Janeiro	188	121	241	404	277	552	3.89	2.48	5.06	3.81	2.62	5.20	-0.02	-0.22	0.22
Rio Grande do Norte	16	11	20	43	30	60	2.22	1.62	2.91	2.67	1.86	3.70	0.20	-0.08	0.55
Rio Grande do Sul	116	81	153	260	174	368	3.59	2.49	4.73	3.51	2.36	4.99	-0.02	-0.27	0.30
Rondônia	03	02	04	12	08	18	1.91	1.37	2.68	2.08	1.43	3.09	0.09	-0.19	0.44
Roraima	01	00	01	03	02	05	2.51	1.84	3.30	2.81	1.99	3.82	0.12	-0.14	0.41
Santa Catarina	34	25	44	106	74	151	2.83	2.12	3.72	3.03	2.13	4.28	0.07	-0.20	0.44
Sergipe	09	06	11	26	18	36	2.20	1.61	2.90	2.89	1.97	4.03	0.31	-0.03	0.72
São Paulo	333	239	433	839	597	1,147	3.41	2.45	4.43	3.39	2.40	4.65	-0.01	-0.22	0.24
Tocantins	03	02	04	11	07	17	1.64	1.12	2.43	2.19	1.45	3.15	0.34	-0.07	0.96

*Age-standardized rate; U.I.: uncertainty interval; all risk factors = physical inactivity, alcohol use, high body-mass index, diet high in sugar-sweetened beverages.

Supplementary Table S4. Number and age-standardized rate (per 100,000 inhabitants) of DALYs from breast cancer due to all risk factors (physical inactivity, alcohol use, high body-mass index, diet high in sugar-sweetened beverages) in women (≥ 25 years old) around the world, Brazil, and Brazilian states in 1990 and 2015.

	**Breast cancer DALYs due to all risk factors**														
	**1990**			**2015**			**1990**			**2015**			**Change (1990-2015)**		
	**DALYs**	**95% U.I.**		**DALYs**	**95% U.I.**		**Rate***	**95% U.I.**		**Rate***	**95% U.I.**		**%***	**95% U.I.**	
Global	1,709,585	1,427,601	1,987,977	2,743,041	2,257,795	3,240,611	85.34	71.13	99.33	75.75	62.29	89.50	-0.11	-0.18	-0.05
Brazil	35,113	26,347	44,419	82,164	60,819	105,915	74.35	56.47	93.90	76.52	56.67	98.55	0.03	-0.09	0.16
Acre	37	25	55	131	81	186	40.92	28.45	61.12	53.01	34.78	73.98	0.30	-0.05	0.75
Alagoas	377	272	498	908	625	1,243	53.81	39.34	71.79	62.38	43.32	85.28	0.16	-0.12	0.54
Amapá	19	12	32	96	55	152	32.16	21.39	55.18	44.66	27.49	69.79	0.39	-0.04	1.01
Amazonas	228	159	308	820	539	1,186	54.69	39.11	72.99	67.17	45.56	97.89	0.23	-0.10	0.67
Bahia	2,046	1,512	2,667	5,245	3,584	7,320	60.29	44.76	78.73	72.51	50.26	100.43	0.20	-0.09	0.57
Ceará	1,256	878	1,656	3,311	2,162	4,573	61.85	43.55	80.66	78.72	52.39	107.85	0.27	-0.04	0.66
Distrito Federal	298	202	407	991	646	1,418	73.80	52.87	99.53	70.19	46.69	99.37	-0.05	-0.30	0.25
Espírito Santo	487	347	637	1,404	980	1,890	61.27	44.78	79.51	67.32	47.11	90.56	0.10	-0.17	0.40
Goiás	679	486	900	2,088	1,439	2,844	61.85	45.97	80.57	65.66	46.25	88.77	0.06	-0.17	0.34
Maranhão	574	388	805	1,538	970	2,259	44.49	30.55	62.39	58.46	37.71	84.79	0.31	-0.09	0.92
Mato Grosso	234	157	318	919	610	1,275	55.35	39.69	73.21	66.36	45.68	91.47	0.20	-0.13	0.59
Mato Grosso do Sul	300	214	395	882	596	1,238	62.69	45.69	80.82	68.58	46.57	96.78	0.09	-0.19	0.43
Minas Gerais	3,514	2,601	4,509	8,356	5,783	11,057	68.14	50.45	87.18	71.03	49.17	93.77	0.04	-0.18	0.36
Paraná	1,805	1,312	2,329	4,705	3,262	6,371	68.97	50.98	89.05	75.22	52.25	101.87	0.09	-0.16	0.40
Paraíba	594	430	769	1,384	940	1,908	54.90	39.88	71.19	69.79	47.30	95.83	0.27	-0.07	0.71
Pará	566	404	752	1,692	1,110	2,466	49.21	36.01	65.26	58.45	39.18	83.46	0.19	-0.13	0.64
Pernambuco	1,633	1,184	2,120	3,711	2,529	5,348	67.96	50.04	87.89	79.83	54.59	113.22	0.17	-0.11	0.54
Piaui	346	245	468	987	677	1,350	47.99	34.27	64.89	64.93	45.21	88.83	0.35	-0.02	0.83
Rio de Janeiro	5,375	3,536	6,960	10,227	6,999	13,923	100.55	65.45	129.94	94.76	65.10	129.08	-0.06	-0.25	0.17
Rio Grande do Norte	435	315	566	1,124	773	1,553	57.18	41.95	73.61	67.14	46.53	92.36	0.17	-0.11	0.51
Rio Grande do Sul	3,280	2,274	4,330	6,323	4,139	8,988	91.62	64.11	120.38	84.55	55.45	120.51	-0.08	-0.31	0.23
Rondônia	105	70	151	366	239	552	50.21	35.80	70.45	53.47	36.30	79.07	0.06	-0.21	0.41
Roraima	20	14	26	99	66	135	63.63	47.17	83.78	67.81	47.11	91.14	0.07	-0.17	0.35
Santa Catarina	998	728	1,306	2,827	1,943	4,019	72.62	54.00	93.79	75.78	52.14	107.85	0.04	-0.23	0.40
Sergipe	250	180	328	708	471	992	57.73	42.03	75.81	72.13	48.89	100.38	0.25	-0.07	0.63
São Paulo	9,566	6,925	12,406	21,002	14,918	28,157	86.64	62.58	112.04	81.13	57.50	108.97	-0.06	-0.25	0.18
Tocantins	92	60	132	320	212	468	43.03	28.72	61.83	55.92	37.44	80.65	0.30	-0.10	0.91

*Age-standardized rate; U.I.: uncertainty interval; all risk factors = physical inactivity, alcohol use, high body-mass index, diet high in sugar-sweetened beverages.

Supplementary Table S5. Number and age-standardized rate (per 100,000 inhabitants) of deaths from all-causes due to physical inactivity in women (≥ 25 years old) around the world, Brazil, and Brazilian states in 1990 and 2015.

	**All-causes mortality due to physical inactivity**														
	**1990**			**2015**			**1990**			**2015**			**Change (1990-2015)**		
	**Deaths**	**95% U.I.**		**Deaths**	**95% U.I.**		**Rate***	**95% U.I.**		**Rate***	**95% U.I.**		**%***	**95% U.I.**	
Global	504,238	393,900	617,657	720,813	572,279	870,134	27.89	21.78	34.17	20.53	16.29	24.79	-0.26	-0.29	-0.24
Brazil	16,093	13,497	18,781	29,806	25,575	34,107	46.48	39.14	54.27	31.03	26.63	35.53	-0.33	-0.37	-0.27
Acre	23	19	28	61	47	76	38.85	31.55	46.87	31.68	24.61	39.58	-0.18	-0.34	0.01
Alagoas	282	231	336	525	407	661	53.87	44.13	63.71	43.00	33.60	53.90	-0.20	-0.35	0.01
Amapá	14	11	18	43	31	57	35.54	28.31	43.44	29.09	21.58	37.53	-0.18	-0.38	0.10
Amazonas	117	95	140	274	212	345	44.40	36.39	53.25	31.60	24.81	39.33	-0.29	-0.43	-0.09
Bahia	1,255	1,027	1,495	2,484	1,961	3,068	46.51	38.24	55.22	37.78	29.91	46.52	-0.19	-0.34	-0.01
Ceará	575	467	692	1,307	1,047	1,631	37.58	30.82	45.14	33.75	27.09	42.02	-0.10	-0.27	0.12
Distrito Federal	88	73	104	268	219	328	37.84	31.61	44.41	20.34	16.66	24.93	-0.46	-0.55	-0.35
Espírito Santo	232	191	277	504	406	625	41.94	34.45	49.83	27.75	22.41	34.38	-0.34	-0.46	-0.19
Goiás	301	249	354	757	613	926	43.15	35.83	50.61	30.64	24.86	37.26	-0.29	-0.41	-0.15
Maranhão	389	311	476	1,013	741	1,334	41.97	34.06	50.95	44.47	32.84	58.02	0.06	-0.19	0.38
Mato Grosso	127	101	153	341	263	428	50.44	40.86	60.59	34.69	27.30	42.87	-0.31	-0.45	-0.14
Mato Grosso do Sul	135	109	164	300	235	370	43.15	34.89	52.08	28.88	22.78	35.39	-0.33	-0.46	-0.17
Minas Gerais	1,699	1,408	2,045	2,869	2,311	3,493	44.80	37.13	53.73	26.59	21.42	32.34	-0.41	-0.50	-0.28
Paraná	849	684	1,011	1,683	1,355	2,091	48.64	39.60	57.69	31.48	25.46	38.94	-0.35	-0.46	-0.22
Paraíba	409	337	491	771	577	990	46.04	38.17	55.01	41.36	31.12	52.97	-0.10	-0.28	0.14
Pará	345	276	424	754	575	986	43.65	35.07	53.30	34.78	26.91	44.76	-0.20	-0.38	0.03
Pernambuco	891	732	1,069.17	1,566	1,222	1,951	48.12	39.53	57.42	38.09	29.92	47.15	-0.21	-0.36	-0.02
Piaui	202	164	244	490	385	610	38.52	31.46	46.51	38.29	30.25	47.45	-0.01	-0.20	0.27
Rio de Janeiro	2,271	1,891	2,682	3,394	2,793	4,051	54.96	45.75	64.63	32.76	26.92	39.10	-0.40	-0.49	-0.30
Rio Grande do Norte	248	206	296	527	417	655	40.33	33.77	48.01	33.94	26.88	42.13	-0.16	-0.30	0.04
Rio Grande do Sul	1,265	1,037	1,501	2,074	1,649	2,615	46.80	38.52	55.55	28.70	22.82	36.21	-0.39	-0.50	-0.23
Rondônia	59	47	72	163	127	207	53.05	43.10	63.07	36.57	28.82	45.67	-0.31	-0.45	-0.14
Roraima	08	07	10	30	24	36	44.64	37.67	52.40	33.15	27.20	39.96	-0.26	-0.39	-0.11
Santa Catarina	434.59	356.10	521.02	893	712	1,114	47.10	38.73	56.23	28.44	22.75	35.34	-0.40	-0.51	-0.24
Sergipe	151	124	180	302	240	383	43.64	36.06	52.17	37.02	29.48	46.51	-0.15	-0.32	0.05
São Paulo	3,665	3,046	4,316	6,241	4,991	7,578	46.45	38.53	54.71	26.50	21.21	32.17	-0.43	-0.51	-0.33
Tocantins	57	43	75	167	128	215	39.57	30.15	50.83	37.39	28.86	47.66	-0.05	-0.29	0.30

*Age-standardized rate; U.I.: uncertainty interval.

Supplementary Table S6. Number and age-standardized rate (per 100,000 inhabitants) of DALYs from all-causes due to physical inactivity in women (≥ 25 years old) around the world, Brazil, and Brazilian states in 1990 and 2015.

	All-causes DALYs due to physical inactivity														
	**1990**			**2015**			**1990**			**2015**			**Change (1990-2015)**		
	**DALYs**	**95% U.I.**		**DALYs**	**95% U.I.**		**Rate***	**95% U.I.**		**Rate***	**95% U.I.**		**%***	**95% U.I.**	
Global	9,630,552	7,534,341	11,771,713	13,620,678	10,855,939	16,511,985	498.69	391.01	609.97	384.61	306.16	466.03	-0.23	-0.19	-0.27
Brazil	393,657	333,020	457,967	655,800	559,298	759,402	916.17	778.02	1,065.96	636.08	542.74	735.13	-0.31	-0.25	-0.35
Acre	613	495	736	1,584	1,248	1,987	762.66	620.05	913.84	666.78	530.17	833.59	-0.13	0.06	-0.29
Alagoas	6,947	5,720	8,258	12,487	9,800	15,694	1,091.05	904.34	1,294.23	901.17	713.13	1,129.77	-0.17	0.03	-0.33
Amapá	350	279	429	1,180	895	1,543	681.25	547.66	833.52	597.18	459.47	775.97	-0.12	0.16	-0.33
Amazonas	3,032	2,515	3,608	7,270	5,793	8,989	847.39	703.13	1,008.24	649.46	518.95	797.59	-0.23	-0.04	-0.38
Bahia	29,695	24,605	35,460	54,547	43,758	67,002	932.17	774.25	1,107.55	775.49	623.29	950.25	-0.17	0.01	-0.32
Ceará	13,134	10,720	15,782	27,947	22,719	34,765	711.55	581.56	851.46	678.08	552.16	843.21	-0.05	0.17	-0.21
Distrito Federal	2,545	2,119	2,983	5,893	4,846	7,276	728.37	609.12	848.71	418.56	345.19	517.23	-0.43	-0.32	-0.51
Espírito Santo	5,906	4,905	7,037	11,492	9,178	14,136	840.21	699.34	998.89	585.21	468.37	718.32	-0.30	-0.16	-0.42
Goiás	8,259	6,885	9,739	19,148	15,617	23,231	872.88	727.77	1,021.98	648.22	531.34	782.38	-0.26	-0.13	-0.37
Maranhão	9,335	7,552	11,317	22,624	16,786	29,418	812.06	665.81	978.24	899.12	670.40	1,162.63	0.11	0.43	-0.14
Mato Grosso	3,642	2,912	4,417	9,124	7,067	11,384	1,003.31	812.22	1,208.67	727.74	568.40	905.82	-0.27	-0.11	-0.42
Mato Grosso do Sul	3,839	3,138	4,605	7,817	6,164	9,630	905.41	744.76	1,078.56	647.30	513.89	795.14	-0.29	-0.13	-0.40
Minas Gerais	41,962	34,679	50,160	63,412	51,282	76,555	894.36	744.86	1,069.83	560.42	453.54	676.26	-0.37	-0.25	-0.47
Paraná	21,534	17,615	25,806	37,683	30,586	46,124	944.30	775.42	1,130.06	639.39	521.84	782.72	-0.32	-0.20	-0.43
Paraíba	9,280	7,686	11,040	16,539	12,596	21,139	905.97	753.14	1,073.72	840.32	642.33	1,072.18	-0.07	0.17	-0.25
Pará	8,396	6,788	10,259	18,824	14,560	24,081	831.14	674.55	1,006.70	708.18	554.03	900.82	-0.15	0.08	-0.33
Pernambuco	21,368	17,539	25,579	35,639	28,051	44,189	968.44	795.04	1,156.22	792.38	625.53	978.47	-0.18	-0.01	-0.33
Piaui	4,918	4,002	5,915	11,200	8,876	13,774	760.70	622.92	909.17	779.41	620.95	954.62	0.02	0.28	-0.16
Rio de Janeiro	57,274	48,151	67,265	74,604	62,083	88,791	1,145.38	962.34	1,343.03	717.47	596.88	853.66	-0.37	-0.28	-0.46
Rio Grande do Norte	5,524	4,598	6,576	11,197	9,004	13,878	770.87	644.58	915.89	682.86	550.23	844.94	-0.11	0.07	-0.26
Rio Grande do Sul	29,317	24,208	34,506	41,296	32,504	51,489	895.24	742.36	1,050.46	573.21	451.24	714.33	-0.36	-0.21	-0.48
Rondônia	1,847	1,490	2,216	4,588	3,614	5,706	1,070.27	875.68	1,273.36	755.47	599.48	934.31	-0.29	-0.13	-0.42
Roraima	240	201	285	895	724	1,099	897.16	757.41	1,054.15	685.72	560.67	832.66	-0.24	-0.10	-0.36
Santa Catarina	10,570	8,700	12,599	19,538	15,666	24,291	888.19	730.39	1,056.85	563.09	454.01	696.33	-0.37	-0.22	-0.48
Sergipe	3,475	2,887	4,138	6,933	5,540	8,733	861.50	716.53	1,026.02	743.95	595.36	931.75	-0.14	0.06	-0.29
São Paulo	89,197	73,757	104,904	128,267	103,934	154,518	896.04	741.80	1,052.10	516.60	419.05	621.42	-0.42	-0.33	-0.50
Tocantins	1,456	1,113	1,868	4,071	3,155	5,139	777.06	600.08	988.52	759.56	590.99	956.26	-0.02	0.29	-0.25

*Age-standardized rate; U.I.: uncertainty interval.
